# The Impacts of Single Preterm Human Donor Milk Compared to Mother’s Own Milk on Growth and Body Composition

**DOI:** 10.3390/nu15071578

**Published:** 2023-03-24

**Authors:** Alexandra Thajer, Esther Teunissen, Theresa Kainz, Elisabeth Calek, Karin Harreiter, Angelika Berger, Christoph Binder

**Affiliations:** 1 Comprehensive Center for Pediatrics, Department of Pediatrics and Adolescent Medicine, Division of Neonatology, Intensive Care and Neuropediatrics, Medical University of Vienna, 1090 Vienna, Austria; 2 Radboud University Medical Center, Radboud University Nijmegen, 6525 Nijmegen, The Netherlands

**Keywords:** mothers’ own milk, human donor milk, single preterm donor milk, preterm infant, growth, body composition, fat-free mass

## Abstract

(1) If mother´s own milk (MOM) is not available, pooled term human donor milk (HDM) is commonly used. Compared to MOM, term HDM contains less protein and fat and is associated with impaired growth. HDM from mothers of preterm infants is an alternative source and contains higher protein levels compared to term HDM, but the impacts on growth and body composition are unclear. (2) Methods: Infants born below 32 weeks of gestation and below 1500 g between 2017–2022, who underwent air displacement plethysmography (Pea Pod^®^) to determine body composition (FFM: fat-free mass; FM: fat mass) at term-equivalent age, were included. A comparison between infants fed with MOM > 50% (MOM-group) and single preterm HDM > 50% (HDM-group) was conducted. (3) Results: In total, 351 infants (MOM-group: *n* = 206; HDM-group: *n* = 145) were included for the analysis. The median FFM-Z-score (MOM-group: −1.09; IQR: −2.02, 1.11; HDM-group: −1.13; IQR: −2.03, 1.12; *p* = 0.96), FM-Z-score (MOM-group: 1.06; IQR: −0.08, 2.22; HDM-group: 1.19; IQR: −0.14, 2.20; *p* = 0.09), and median growth velocity (MOM-group: 23.1 g/kg/d; IQR: 20.7, 26.0; HDM: 22.5 g/kg/d; IQR: 19.7, 25.8; *p* = 0.15) values were not significantly different between the groups. (4) Conclusion: Single preterm HDM is a good alternative to support normal growth and body composition.

## 1. Introduction

Mother´s own milk (MOM) is considered the gold standard for preterm infants’ nutrition [1,2]. It contains bioactive proteins, lipids, oligosaccharides, and immunomodulatory components and has essential benefits on preterm infants’ health and neurodevelopment [3,4]. If MOM is not available or is insufficient, pasteurized pooled term human donor milk (HDM) is the second best nutritional source for preterm infants [5]. Preterm formula is less commonly used in neonatal intensive care units (NICUs), as several trials have demonstrated that preterm formula compared to human milk increases the risk for late-onset sepsis, necrotizing enterocolitis (NEC) [6,7,8], and bronchopulmonary dysplasia (BPD) [9]. The nutritional diet is essential for adequate growth and body composition in preterm infants [2,10]. The nutrient content of human milk is highly variable during the lactation period and depends on several factors, including the gestational age at delivery and lactation stage [11]. Several studies stated that postnatal growth restriction is associated with retinopathy of prematurity (ROP), BPD, NEC [12,13], and impaired neurodevelopment [14,15]. Assessment of the body composition are valuable tools to evaluate the nutritional status [16], and particularly the fat-free mass (FFM), which is a good prognostic parameter for neonatal neurodevelopment [17,18]. Studies have shown that a higher FFM at term-equivalent age is linked to better brain growth and neurodevelopment [19,20]. Therefore, the nutritional goal is to avoid postnatal growth failure and loss of FFM. The most common practice in NICUs to date is to use pooled term HDM if MOM is not available [21]. Pooled term HDM is obtained from different mothers of infants born at term as well as from different stages of lactation. Studies described that pooled term HDM shows a different composition than MOM from a preterm infant [22,23]. The macronutrients, particularly the protein and energy levels, which are essential for adequate growth and neurodevelopment, were significantly lower in pooled term HDM compared to MOM [11,23]. Furthermore, previous studies stated that pooled term HDM compared to MOM has a negative impact on growth in preterm infants [24,25]. However, single preterm HDM from a mother of preterm infants is an alternative source for preterm infants’ nutrition. Single preterm HDM compared to pooled term HDM contains higher concentrations of proteins [23,26]. It is well known that protein and energy intakes are associated with better growth [27,28]. Consequently, single preterm HDM for preterm infants’ nutrition might be a good alternative to support adequate growth, body composition, and development. However, single preterm HDM is not commonly used in NICUs, and the possible positive effects on growth and body composition have not been investigated so far. Therefore, the aim of this study was to evaluate the impacts of single preterm HDM in comparison to MOM on growth and body composition at term-equivalent age in preterm infants.

## 2. Materials and Methods

### 2.1. Study Design

This retrospective study was conducted at a level IV neonatal intensive care unit between the years 2017 and 2022 at the Department of Pediatrics and Adolescent Medicine of the Medical University of Vienna, Austria. The study was approved by the local ethics committee (no. 1285/2022). The aim of the study was to evaluate the impacts of MOM in comparison to single preterm HDM for enteral nutrition in preterm infants on growth and body composition at term-equivalent age.

### 2.2. Patient Groups

Preterm infants born below 32 weeks of gestation and with a birth weight below 1500 g during 2017 and 2022, who underwent air displacement plethysmography (Pea Pod^®^) to determine body composition (FFM and FM (fat mass)) at term-equivalent age, were included for the analysis. In our hospital, body composition measurements are performed in all preterm infants born below 32 weeks of gestation at term-equivalent age as part of our standard clinical practice. A comparison between preterm infants fed predominately with MOM > 50% (MOM-group) and infants fed predominately with single preterm HDM > 50% (HDM-group) of the total volume of milk intake during hospital stay was conducted. A cut-off of 50% was used according to the current literature and to assure adequate comparability [24,29]. Infants with genetic and metabolic diseases with primary effects on growth and body composition were excluded from the analysis. The mode of delivery was chosen in accordance with the underlying medical condition leading to the preterm delivery. As this cohort consisted of extremely preterm infants only and the induction of labor at this gestational age is insufficient, the majority of preterm infants were delivered by cesarean section according to the local protocol.

### 2.3. Body Composition

The preterm infants underwent non-invasive air displacement plethysmography (Pea Pod, COSMED, Concord, CA, USA) to determine their body composition at term-equivalent age (between 37 and 47 weeks of postmenstrual age). The device is based on a two-compartment model of the body composition, including FFM and FM, and uses the inverse relation between the pressure and volume to derive the body volume for a subject [30]. The accuracy and reliability of the device has been reported previously [31]. The FFM and FM-Z-scores were calculated according to the published sex and gestational-age-specific reference charts for premature infants [32]. The FFM and FM percentage, FFM and FM-Z-scores, and FFM and FM kilogram weights are reported.

### 2.4. Nutitional Management

Enteral nutrition was commenced directly after birth using pasteurized MOM or pasteurized single preterm HDM and increased gradually according to the local protocol (maximum 20 mL/kg per day). The MOM and preterm HDM were pasteurized using holder pasteurization at 64 degrees for 30 min. Mothers of preterm infants with a birthweight below 1500 g and below 32 weeks of gestational age who were admitted to our hospital were asked by our lactation team to donate breastmilk for other preterm infants. The donating mothers were identified by our milk kitchen team due to having a full breastmilk stock, which is defined as a stock of approximately 5 L and sufficient to feed their own infant. All donating mothers underwent a health and serological check for the human immunodeficiency virus, hepatitis A, hepatitis B, hepatitis C, cytomegalovirus, toxoplasmosis, and rubella. After pasteurization, the milk from each mother was portioned in 50 mL or 100 mL bottles and each bottle was used for a single preterm infant. A small sample (1 mL) of each bottle was sent to the Austrian agency for health and food safety for microbiological quality checks. If single preterm HDM was used, the infants were switched to preterm formula after 32 weeks of postmenstrual age. The milk feedings were fortified when 100 mL/kg of enteral nutrition was reached (bovine-based fortifier or human milk-based fortifier). The infants received parenteral nutrition directly after birth and lipid emulsions were started at 1.0 g/kg/d, protein at 1.5 g/kg/d, and carbohydrates at 5.6 mg/kg/min. The lipids and proteins were increased daily by 0.5 g/kg/d and dosed up to 3.5 g/kg/day at the discretion of the attending physicians, and were reduced in relation to enteral nutrition. The carbohydrates were increased daily according to the blood glucose levels (maximum 12 mg/kg/min). The parenteral nutrition was stopped at 140–160 mL/kg/d of enteral nutrition. The enteral and parenteral nutrition was carried out according to the ESPGHAN recommendations [2,33]. The time on parenteral nutrition in days and total volumes in mL of MOM, single preterm HDM, and preterm formula are reported.

### 2.5. Statistics

The demographic data and descriptive statistics are expressed as median and interquartile range (IQR) values and frequency distributions. Weight, length, head circumference, FFM, and FM values were transformed into Z-scores using the LMS method, based on sex- and gestational-age-specific growth charts [32]. A sample size of 286 infants (143 infants for each group) was calculated to detect a 0.4% difference in FFM-Z-scores (primary outcome parameter) between the groups (−1.4 versus −1.0 Z-scores; SD −1.2; level of significance 0.05, two-tailed, power 80%). A multivariable regression model was used to examine the association between the FFM-Z-scores (primary outcome parameter), FM-Z-scores, and growth parameters (weight, length, head circumference) at discharge (secondary outcome parameters) and the study groups (MOM-group and HDM-group) with adjustment for the covariates, namely the sex [34], gestational age at birth [35], birth weight Z-score [36], gestational age, and length at measurement [35] and time on parenteral nutrition [37]. A multivariable regression model was used to examine the associations between FFM-Z-scores, FM-Z-scores, and growth parameters (weight, length, head circumference) at term-equivalent age and the total enteral intake of MOM, HDM, and formula with adjustment for the covariates, namely the sex, gestational age at birth, birth weight Z-score, gestational age, and length at measurement and time on parenteral nutrition. Pearson’s correlation test was used to assess the correlation between the FFM-Z-score and FM-Z-score, anthropometric parameters (weight, length, and head circumference), and the total MOM and total formula intake. Mann–Whitney U and Pearson qui-square tests were used to compare the baseline characteristics, growth velocity (grams/kg/day from birth until discharge), and co-morbidities of the two groups (MOM-group and HDM-group). The data were analyzed using SPSS version 28 for Mac (IBM Corp, Armonk, New York, NY, USA). A *p*-value of < 0.05 was considered statistically significant [38].

## 3. Results

In total, 351 preterm infants (MOM-group: *n* = 206; HDM-group: *n* = 145) were included for the analysis. The baseline characteristics, prenatal data, growth velocity, and short-term outcome parameters are shown in Table 1. The gestational age (MOM-group: median 27.2; IQR: 25 + 6, 28 + 6 weeks + days; HDM-group: median 27 + 0, IQR: 25 + 2, 28 + 4 weeks + days) (*p* = 0.10) and anthropometric parameters (weight, length, and head circumferences) at birth were not significantly different between the study groups (Table 1). The median gestational ages at discharge were not significantly different between the groups (MOM-group: median: 38 + 3; IQR: 37 + 1, 40 + 2 weeks + days; HDM-group: median: 38 + 3, IQR: 37 + 2, 39 + 4 weeks + days) (*p* = 0.77). The short-term outcome parameters and anthropometric parameters (weight, length, and head circumferences) at discharge were not significantly different between the groups (Table 1). The median weight growth velocity from birth until discharge was not significantly different in the MOM-group (23.1, IQR: 20.7, 26.0 g/kg/day) compared to the HDM-group (22.5, IQR: 19.7, 25.8 g/kg/d) (*p* = 0.15). The median increases in length and head circumference from birth until discharge were not significantly different between the groups (*p* = 0.06, *p* = 0.20, respectively) (Table 1).

The parameters of the enteral and parenteral nutrition are displayed in Table 2. The days on parenteral nutrition (MOM-group: median: 17, IQR: 12, 20 and HDM-group: median: 18, IQR: 14, 21) were not significantly different between the groups (*p* = 0.13). As expected, the total enteral intake for MOM was significantly higher in infants in the MOM-group (*p* < 00.1) and the total enteral intake of preterm single HDM was significantly higher in infants in the HDM-group (*p* < 0.001). The total enteral formula intake was significantly higher in infants in the HDM-group compared to infants in the MOM-group (*p* < 0.001). Here, 69.9% of preterm infants (*n* = 144/206) in the MOM-group were fed exclusively MOM at discharge. The median age when formula feeding was implemented in the HDM-group was 32 + 5 weeks + days (IQR: 32 + 3, 33 + 2 weeks + days).

The unadjusted anthropometric parameters (weight, length, and head circumference) at the time of measurement and body composition parameters (FFM and FM) are shown in Table 3. The linear regression analysis revealed that the enteral nutritional intake (MOM-group versus HDM-group) had no significant impact on the FFM-Z-scores (*p* = 0.96) and FM-Z-scores (*p* = 0.09) at term-equivalent age. Furthermore, the linear regression analysis showed that the enteral nutritional intake (MOM-group and HDM-group) had no significant impact on the weight (*p* = 0.45), length (*p* = 0.29) and head circumference (*p* = 0.67) at term-equivalent age. The linear regression showed that the total enteral nutrition (MOM, HDM, and formula) had no significant influence on the FFM-Z-score (*p* = 0.62, *p* = 0.66, *p* = 0.16; respectively), FM-Z-score (*p* = 0.76, *p* = 0.52, *p* = 0.86; respectively), weight (*p* = 0.40, *p* = 0.59, *p* = 0.15; respectively), length (*p* = 0.75, *p* = 0.83, *p* = 0.22; respectively), and head circumference at measurement (*p* = 0.48, *p* = 0.15, *p* = 0.47; respectively). The total MOM intake, FFM-Z-score (*p* = 0.45), and FM-Z-score (*p* = 0.62) showed no significant correlations. The total MOM intake and weight (*p* = 0.16), length (*p*= 0.14), and head circumference (*p* = 0.34) at measurement revealed no significant correlations. The total formula intake, FFM-Z-score (*p* = 0.89), and FM-Z-score (*p* = 0.27) showed no significant correlations. The total formula intake and weight (*p* = 0.15), length (*p* = 0.75), and head circumference (*p* = 0.39) at measurement revealed no significant correlations.

## 4. Discussion

This study showed that the body composition (FFM-Z-score and FM-Z-score) and anthropometric parameters (weight, length, and head circumference) at term-equivalent age were not significantly different in preterm infants who received primarily MOM compared to infants who received primarily single preterm HDM. The growth velocity, length, and head circumference increases from birth until discharge were not significantly different between the groups. These data underline that single preterm HDM is a good alternative to MOM and supports normal growth and body composition at term-equivalent age, which is a good prognostic parameter of neurodevelopmental outcome.

MOM is the best and most optimal nutritional source for preterm infants, and if not available or if it is insufficient then HDM is recommended [21]. In general, the use of pasteurized term HDM is increasing in NICUs and has been associated with higher rates of exclusive human milk feeding at discharge [5,39]. However, several studies have demonstrated that the macronutrients, particularly the protein levels, were significantly lower in pooled term HDM compared to MOM [5,22,23]. It is well accepted that adequate protein and energy intakes are essential for normal growth [28,40]. Growth is usually monitored by anthropometric parameters (growth, length, and head circumference), but studies have shown that body composition measurements, particularly the assessment of FFM, are additional valuable tools to evaluate the nutritional status and optimal growth [20,41]. In a previous study, we showed that the FFM-Z-score is a better benchmark for optimal brain growth than anthropometric parameters [19]. Furthermore, studies provide evidence that deficits in FFM are associated with neurodevelopmental impairment [18,20]. Therefore, the major nutritional goal is to achieve an optimum nutritional intake to avoid postnatal growth restrictions and especially loss of FFM. However, several trials have stated that pooled term HDM in comparison to MOM has a negative impact on growth [24,42,43]. Browell et al. [42] evaluated the relationship between MOM, pooled term HDM, and formula on growth rates in around 300 preterm infants. The study showed that the weight and head circumference velocity significantly decreased by 0.17 g/kg/day for every 10% increase in HDM intake during the hospital stay [42]. Furthermore, Montjaux-Regis et al. [43] reported that the growth velocity was significantly lower in infants fed with pooled HDM in comparison to infants fed with MOM. In conclusion, poorer growth with pooled term HDM might be related to the inadequate nutrient composition of HDM in comparison to MOM. Further studies have evaluated the impact of pooled term HDM compared to MOM on body composition. Piemontese et al. [25] reported that preterm infants being fed predominantly with MOM (>50% of the total enteral intake) have higher FFM values—as measured by the body composition at term-equivalent age—than infants fed with <50% MOM of the total enteral intake. These data show that the macronutrient content in pooled term HDM might be inadequate to support normal growth and body composition, which are essential for brain growth and development [25,43].

Single preterm HDM is an alternative source of preterm infants’ nutrition and contains higher concentrations of protein than pooled term HDM [23,42]. Consequently, single preterm HDM might be an valuable alternative to pooled term HDM for preterm infant nutrition to support adequate growth and body composition [39]. In a previous study, we stated that the use of single preterm HDM is feasible and has a positive effect on the time to full enteral feeding, ROP, and culture-proven sepsis in comparison to infants fed with preterm formula [44]. Furthermore, the growth parameters were not significantly different between the groups [44]. However, the impacts on single preterm HDM compared to MOM on growth, and especially on body composition, have not been investigated so far. In our study, the anthropometric parameters at discharge, growth velocity, and median FFM-Z-score and FM-Z-score were not significantly different between infants fed primarily with MOM compared to infants fed primarily with single preterm HDM. In comparison to previous published studies, the median FFM-Z-score (MOM-group: −1.09; HDM-group: −1.13) and median FM-Z-score (MOM-group: 1.06; HDM-group: 1.19) at term-equivalent age and median growth velocity from birth until discharge (MOM-group: 23.1; HDM-group: 22.5 g/kg/day) were in normal ranges in both group [32,41]. However, the preterm infants in the HDM-group received significantly more formula for enteral nutrition (around 5 L) compared to infants in the MOM-group (around 1 L), which might influence the growth and body composition at term-equivalent age. The linear regression model showed that the source of enteral nutritional intake had no significant impact on growth or body composition. Additionally, the formula intake and growth, as well as the body composition, showed no significant correlation. These results are supported by the systematic review and meta-analysis by Strobel et al. [6], showing that formula in comparison to MOM had no significant effect on growth in preterm infants.

We hypothesized that the higher macronutrient composition, especially the protein content, in single preterm HDM in comparison to pooled term HDM positively effects growth and body composition. Preterm infants who received single preterm HDM might receive an adequate nutritional supply to support normal growth and body composition. This hypothesis is supported by a meta-analysis by Gidrewicz et al. [23] stating that the protein levels were higher in HDM from mothers of preterm infants compared to pooled HDM from mothers of term infants. The protein levels differed significantly within the first two weeks of gestation and were very similar thereafter [23]. In our study, we did not assess the macronutrient contents of MOM and single preterm HDM, meaning that drawing a causal relationship was not possible due to the nature of the study. Furthermore, it is well known that the amount of total enteral intake, gestational age at birth, and postnatal age influence the protein content [24,29]. In our study, the baseline characteristics of the two study groups and the ages at measurement were not significantly different, meaning a bias was unlikely. However, we showed that the growth and body composition at term-equivalent age were not significantly different in preterm infants who received primarily MOM compared to infants who received primarily single preterm HDM and formula starting at 32 weeks of gestation until the body composition measurement. Further prospective, blinded, and randomized controlled trials are necessary to draw firm conclusions. In summary, we showed that single preterm HDM compared to MOM had no negative impact on growth and particularly on body composition, which is a good parameter for brain growth and neurodevelopment. These data support the hypothesis that the macronutrient content in preterm single HDM is sufficient to support normal growth and body composition.

One limitation of the study is that data on long-term growth and neurodevelopment are not yet available. Follow-up data on growth and long-term neurodevelopment will be assessed using the Bayley Scales of Infant and Toddler Development at the age of 2 and 3 years of age and the Kaufman–Assessment Battery of Children at the age of 5.5 years of age. Furthermore, the macronutrient contents of MOM and single preterm HDM were not assessed. However, previous studies demonstrated that HDM from mothers of preterm infants contains significantly higher protein levels than MOM of term infants [23]. A strength of our study is the use of body composition to quantify qualitative growth at term-equivalent age. Air displacement plethysmography is based on a two-compartment model and calculates body fat values from the body weight and volume [45]. The method provides reliable and accurate assessments of body fat in comparison to the most robust and sensitive four-compartment model [45]. The limitation of the method is the underlying assumption of the constant hydration of FFM, which varies with age and physiological status [45,46]. Previous studies stated that the biological variability of FFM is relatively low (lower than 0.55%) in preterm infants [47]. Furthermore, the baseline characteristics were well balanced and the median ages at measurement were not significantly different between the groups, meaning a bias from the method seems unlikely. Another strength is the prespecified retrospective sample size analysis with a large sample size of around 350 preterm infants. To the best of our knowledge, this is the first study evaluating the effects of single preterm HDM compared to MOM on growth and body composition.

## 5. Conclusions

This study found that the body composition (FFM-Z-score and FM-Z-score; *p* = 0.96, *p* = 0.09, respectively) and anthropometric parameters (weight, length, and head circumference; *p* = 0.45, *p* = 0.29, *p* = 0.67, respectively) at term-equivalent age were not significantly different in preterm infants who received primarily MOM compared to infants who received primarily single preterm HDM. The growth velocity values (weight, length, and head circumference) from birth until discharge were not significantly different between the groups (*p* = 0.15, *p* = 0.06, and *p* = 0.20, respectively). These data underline the hypothesis that the macronutrient content in single preterm HDM is adequate to support normal growth and especially body composition, which is a good marker for brain growth and neurodevelopment.

## Figures and Tables

**Table 1:** Baseline characteristics, growth velocity, and short-term outcome parameters.

Variables	MOM-Group (*n* = 206)	HDM-Group (*n* = 145)	*p*-Values
Gestational age, weeks-median (IQR)	27 + 2 (25 + 6, 28 + 6)	27 + 0 (25 + 2, 28 + 4)	0.10
Male, (*n*, %)	113 (55)	81 (56)	0.46
Birth weight, gram-median (IQR)	960 (725, 1150)	870 (670, 1110)	0.09
Birth weight, Z-score-median (IQR)	0.0 (−0.9, 0.9)	−0.7 (−1.2, −1.0)	0.07
Birth length, cm-median (IQR)	35 (32, 38)	34 (32, 37)	0.08
Birth length, Z-score-median (IQR)	0.2 (−1.0, 1.0)	−0.4 (−1.1, 0.1)	0.14
Head circumference, cm-median (IQR)	25 (23, 27)	24 (23, 26)	0.11
Head circumference, Z-score-median (IQR)	0.6 (−0.8, 1.4)	0.0 (−0.5, 1.3)	0.38
Antenatal steroids for lung maturation (*n*, %)	178 (86)	139 (90)	0.36
PPROM, (*n*, %)	66 (32)	45 (31)	0.84
Cesarean section (*n*, %)	190 (92)	133 (92)	0.86
Small for gestational age (*n*, %)	33 (16)	20 (14)	0.57
Umbilical artery pH-median (IQR)	7.31 (7.28, 7.36)	7.32 (7.27, 7.36)	0.92
Apgar Score, 5 min-median (IQR)	9 (8, 9)	9 (8, 9)	0.07
Apgar Score, 10 min-median (IQR)	9 (9, 9)	9 (9, 9)	0.15
ROP grade II–IV (*n*, %)	19 (9)	14 (10)	0.89
IVH grade III–IV (*n*, %)	5 (2)	3 (2)	0.82
Chronic lung disease, (*n*, %)	21 (10)	10 (7)	0.28
NEC stage ≥ 2, (*n*, %)	13 (6)	7 (5)	0.55
Postmenstrual age at discharge, week-median (IQR)	38 + 3 (37 + 1, 40 + 2)	38 + 3 (37 + 2, 39 + 4)	0.77
Weight at discharge, gram-median (IQR)	2807 (2508, 3090)	2760 (2407, 3045)	0.29
Weight velocity, g/kg/d—from birth until discharge	23.1 (20.7, 26.0)	22.5 (19.7, 25.8)	0.15
Length at discharge, gram-median (IQR)	46.0 (44.2, 48.0)	45.5 (43.0, 48.5)	0.09
Length increase, cm/week-from birth until discharge	1.02 (0.88, 1.14)	0.95 (0.81, 1.09)	0.06
HC at discharge, gram-median (IQR)	33.0 (32.0, 34.0)	33.0 (31.5, 34.0)	0.07
HC increase, cm/week-from birth until discharge	0.75 (0.67, 0.83)	0.73 (0.61, 0.88)	0.20

Preterm premature rupture of the membrane (PPROM); retinopathy of prematurity (ROP); intraventricular hemorrhage (IVH); necrotizing enterocolitis (NEC), head circumference (HC); interquartile range (IQR).

**Table 2:** Parameters of enteral and parenteral nutrition.

Variables	MOM-Group (*n* = 206)	HDM-Group (*n* = 145)	*p*-Values
Days on any parenteral nutrition *	17 (12, 20)	18 (14, 21)	0.13
Enteral nutrition			
Mother´s own milk, total volume in liters *	13.71 (9.66, 16.81)	1.52 (0.40, 3.58)	<0.001
Preterm human donor milk, total volume in liters *	0.35 (0.07, 1.18)	10.23 (8.79, 11.83)	<0.001
Preterm formula, total volume in liters *	0.80 (0.57, 1.05)	4.90 (3.56, 6.78)	<0.001
Exclusively mother´s own milk at discharge, (*n*, %)	144 (70%)	-	-

* Median and interquartile range (IQR).

**Table 3:** Unadjusted anthropometric parameters and body composition parameters at term-equivalent age.

Unadjusted Variables	MOM-Group (*n* = 206)	HDM-Group (*n* = 145)
Postmenstrual age at measurement	42 + 1 (39 + 6, 44 + 6)	43 + 2 (40 + 1, 45 + 6)
Body composition parameters		
FFM, percentage	78.7 (73.5, 84.3)	77.0 (72.5, 82.7)
FM, percentage	21.3 (15.8, 26.4)	23.0 (17.4, 27.6)
FFM, gram	2770 (2400, 3311)	3060 (2400, 3705)
FM, gram	741 (498, 1109)	854 (578, 1313)
FFM, Z-score	−1.09 (−2.02, 1.11)	−1.13 (−2.03, 1.12)
FM, Z-score	1.06 (−0.08, 2.22)	1.19 (−0.14, 2.20)
Anthropometric parameters		
Weight, gram	3528 (3002, 4479)	4025 (3044, 5032)
Length, cm	50.5 (48.0, 54.0)	51.0 (48.8, 55.1)
Head circumference, cm	35.3 (34.0, 37.0)	35.7 (34.0, 37.6)
Data are median and Interquartile Range (IQR)		

## Data Availability

The data presented in this study are available on request from the corresponding author. The data are not publicly available due to ongoing research.
